# Methamphetamine Inhibits Long-Term Memory Acquisition and Synaptic Plasticity by Evoking Endoplasmic Reticulum Stress

**DOI:** 10.3389/fnins.2020.630713

**Published:** 2021-01-14

**Authors:** Guang Chen, Xiaoning Wei, Xiang Xu, Gang Yu, Zheng Yong, Ruibin Su, Luyang Tao

**Affiliations:** ^1^Department of Forensic Medicine, Medical School of Soochow University, Suzhou, China; ^2^Becton, Dickinson and Company, Guangzhou, China; ^3^School of Forensic Medicine, Wannan Medical College, Wuhu, China; ^4^State Key Laboratory of Toxicology and Medical Countermeasures, Beijing Institute of Pharmacology and Toxicology, Beijing, China

**Keywords:** methamphetamine, neurotoxicity, endoplasmic reticulum stress, memory, tauroursodeoxycholic acid

## Abstract

Methamphetamine (MA), an illicit drug abused worldwide, leads to cognitive impairment and memory loss. However, the detailed mechanisms of MA-induced neurologic impairment are still unclear. The present study aimed to investigate the mechanisms of MA-induced inhibition of memory acquisition from the perspective of endoplasmic reticulum (ER) stress. ER stress, caused by the accumulation of wrongly folded proteins in the ER, is important for new protein synthesis, which further influence the formation of long-term memory. A subacute MA poisoning model of mice was established and several behavioral experiments were performed, including elevated plus maze, Morris water maze, electro-stimulus Y-maze, and novel object recognition tasks. The present results suggested that 4 days exposure to MA induced significant memory loss. Whereas, this damage to memory formation could be protected when mice were pre-treated with ER stress inhibitor, tauroursodeoxycholic acid (TUDCA). The results of Western blotting showed that subacute exposure to MA increased the expression levels of ER stress marker proteins, such as binding immunoglobulin protein, phosphorylated eukaryotic translation initiation factor 2α, cyclic AMP-dependent transcription factor (ATF)-4, ATF-6, and CCAAT-enhancer binding protein homologous protein. Meanwhile, the enhanced expression levels of these proteins were reversed by TUDCA, indicating that MA administration induced memory loss by evoking ER stress in the hippocampus. We also found that MA inhibited the induction of long-term potentiation (LTP) in the hippocampus. Nevertheless, LTP could be induced when mice were pre-treated with TUDCA. In conclusion, MA inhibited long-term memory acquisition and synaptic plasticity via ER stress.

## Introduction

Methamphetamine (MA), an amphetamine-type stimulant, has been abused worldwide and its use has increased the heath and economic burden to individuals and society (Courtney and Ray, 2014; Xu et al., 2019). Acting mainly on the dopaminergic, noradrenergic, and serotonergic pathways of the central nervous system (Cruickshank and Dyer, 2009), MA induces significant neurotoxicity (Shaerzadeh et al., 2018), one of which is cognitive impairment. Clinical researchers have found that chronic MA abuse leads to cognitive dysfunction (Scott et al., 2007), which is related to attention, psychomotor speed, and executive function in addition to memory loss. It is also suggested by previous studies that MA-treated rodents exhibit deficits in spatial and recognition memory (Belcher et al., 2008; North et al., 2013). In contrast, MA-induced memory enhancement has also been detected in rodents (Cao et al., 2013). Collectively, the differing effects of MA on memory may be attributed to different models of MA administration and behavioral tasks carried out in different studies.

Oxidative stress is considered as one important mechanism of MA-induced neurotoxicity that causes nerve cell death by disrupting cellular organelle function, including that of the endoplasmic reticulum (ER) (Krasnova and Cadet, 2009; Yu et al., 2015). Induced by the accumulation of unfolded proteins and protein aggregation, ER stress is initiated by phosphorylated inositol requiring kinase 1α (p-IRE1α), PKR-like ER kinase (PERK) or cyclic AMP-dependent transcription factor-6 (ATF-6). ER stress is mainly mediated by three pathways (Kim et al., 2008) and the role of ER stress is to reduce the protein load of the ER and promote the degradation or reassembly of misfolded proteins. However, if ER stress is too severe or lasts too long, it can lead to cell death (Sano and Reed, 2013). Recent studies have found that ER stress is involved in the pathophysiology of several diseases, including neurodegeneration, cancer, diabetes, stroke, and inflammation (Oyadomari and Mori, 2004). MA can also induce ER stress and lead to cell death via ER stress-associated autophagy and apoptosis (Jayanthi et al., 2004). However, it is still unclear whether ER stress is involved in MA-induced memory loss. As protein synthesis is essential for long-term memory induction, it can be postulated that ER stress may be involved in MA-induced long-term memory impairment.

In the present study, a subacute administration method of MA, which has been found to induce ER stress and neurotoxicity in mice (Cai et al., 2016), was used and different behavioral experiments, including elevated plus maze, Morris water maze, electro-stimulus Y-maze, and novel object recognition tasks, were conducted to investigate the impairment of memory caused by MA. Next, tauroursodeoxycholic acid (TUDCA), a general inhibitor for ER stress, was used to inhibit ER stress and the effect of TUDCA pre-treatment on MA-induced memory loss was evaluated. TUDCA, the taurine-conjugated derivative of ursodeoxycholic acid (UDCA) is a bile acid synthesized by intestinal bacteria, and used for the treatment of cholestatic liver diseases, diabetes, and atherosclerosis (Lebensztejn, 2000; Ozcan et al., 2006). Recent studies have found that TUDCA alleviates ER stress by enhancing ER folding ability and preventing protein aggregation (Liu et al., 2015). Meanwhile, we investigated the effect of ER stress on the synaptic plasticity by evaluating the role of ER stress in the induction of long-term potentiation (LTP) in the hippocampus, which is closely associated with memory formation and storage. LTP is defined as a long-term increase in synaptic response following a high frequency stimulation (HFS) (Baltaci et al., 2019). It has been suggested that LTP is not only a laboratory phenomenon, but also involved in the information storage (Pastalkova et al., 2006).

## Materials and Methods

### Drugs

MA was obtained from the Beijing Institute of Pharmacology and Toxicology. TUDCA was purchased from Shanghai Aladdin Bio-Chem Technology Co., Ltd. (cat. no. S101371). All drugs were dissolved in the saline (0.9% NaCl solution) and prepared to a specified concentration. The mice were divided into four groups: (i) Saline, (ii) TUDCA, (iii) MA, and (iv) TUDCA + MA. In the MA group, mice were administered intraperitoneal (i.p.) injections of 15 mg/kg MA (twice a day) for 4 days. In the TUDCA + MA group, mice received i.p. injections of 200 mg/kg TUDCA 60 min before receiving MA injections every time. In the Saline and TUDCA groups, mice were administered i.p. injections of the saline and 200 mg/kg TUDCA, respectively.

### Animals and Housing

Male C57BL/6 mice aged 6–8 weeks were obtained from SPF (Beijing) Biotechnology Co., Ltd. Mice had free access to water and food in standard experimental housing with a constant temperature range (25°C) and a 12 h light/dark cycle. All experimental animal procedures were conducted in accordance with the National Institutes of Health (NIH) and were approved by the Animal Care and Use Committee of the Beijing Institute of Pharmacology and Toxicology. Different mice were used in each of the following experiments.

### Elevated Plus Maze

**FIGURE 1:**
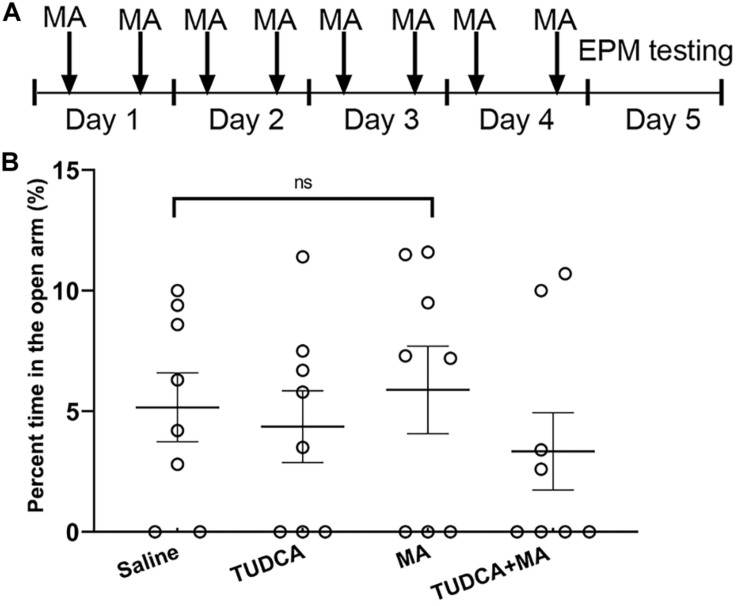
MA administration has no effect on the anxiety-like behavior of mice. **(A)** The experimental flow diagram of elevated plus maze task. **(B)** There was no significant difference in the percent time spent in the open arms between the four groups. Data was presented as the mean ± SEM, *n* = 8 per group, ns indicated *p* > 0.05.

Elevated plus maze (EPM) task was initially performed (*n* = 8 per group) to evaluate whether drug administration had an effect on animal anxiety-like behavior, using a standard experimental apparatus manufactured by Shanghai Jiliang Software Technology Co., Ltd. The apparatus consisted of two open arms (30 × 5 cm, length × width) and two closed arms (30 × 5 × 15 cm, length × width × height) elevated 50 cm above the floor. The EPM testing was conducted as previously described (Psotta et al., 2015). At the beginning of the experiment, mice were placed in the central zone of the maze facing an open arm and were allowed to freely explore the maze for 5 min. The maze was carefully cleaned after each trial to remove odor cues. EPM testing was performed 24 h after the final injection of drugs (Figure 1A). Increased anxiety-like behavior was indicated by a lower percent time in the open arms.

### Morris Water Maze

**FIGURE 2:**
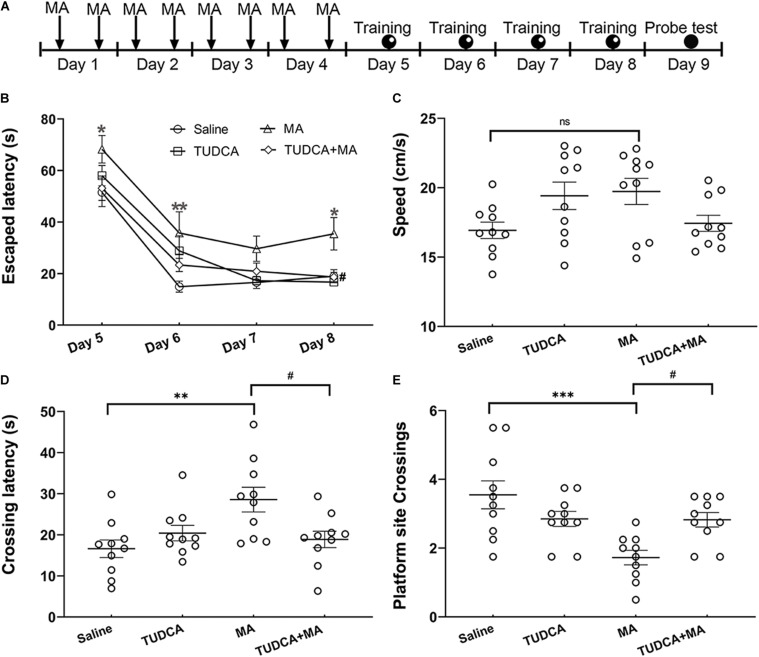
MA-induced deficits of spatial memory are protected by TUDCA. **(A)** The experimental flow diagram of Morris water maze task. **(B)** MA-treated mice exhibited increased escaped latencies on days 1, 2, and 4 during the training session, in comparison with that of saline-treated mice. The escaped latency of TUDCA + MA-treated mice was less than that of MA-treated mice on day 4. **(C)** There was no difference in swimming speed between the four groups in the probe test. (**D**) MA-treated mice exhibited increased crossing latency, compared with that of saline-treated mice in the probe test, whereas mice of the TUDCA + MA group exhibited decreased latency, compared with that of MA-treated mice. **(E)** The platform site crossings of MA group were less than the Saline group, while mice of the TUDCA + MA group exhibited more platform position crossings than the MA group. *p < 0.05, **p < 0.01, ***p < 0.001 vs. the Saline group. ^#^*p* < 0.05 vs. the MA group, ns indicated *p* > 0.05. Data was presented as the mean ± SEM, *n* = 10 per group.

The Morris water maze (MWM) task was performed (*n* = 10 per group) to evaluate the effect drug administration on the spatial memory formation of mice. The apparatus, which was manufactured by Shanghai Jiliang Software Technology Co., Ltd., consisted of a stainless-steel circular tank (120 cm diameter, 60 cm height) filled with 23 ± 1°C water to a depth of 40 cm. A Plexiglas platform (10 cm diameter) was submerged 1.5 cm below the surface of the water during the training session and distal cues were placed within the experimental room. The protocol of MWM task was according to the previous reports (Vorhees and Williams, 2006; Cao et al., 2013) with some modifications. Mice were administered i.p. injections of drugs for 4 days followed by 4 days training with testing conducted 24 h after the last training session (Figure 2A). During the training session, mice were held facing the tank wall and randomly placed into the pool from one of four fixed entry points and allowed to swim freely for 90 s. A trial ended when the mouse climbed onto the platform and stopped for 5 s or when the 90 s time limit had elapsed. Mice were given a 15 min rest between two consecutive trials. A 60 s probe testing was performed 24 h after the last day of training with the platform removed. Swimming tracks were recorded and analyzed. Escaped latency (i.e., the interval between the start of the experiment and when mice climbed onto the platform) was used to evaluate spatial learning during the training stage. During the testing, platform site crossings and crossing latency (i.e., the interval between the start of the test and when mice swam across the site of the platform) were used to evaluate spatial memory retrieval.

### Electro-Stimulus Y-Maze

**FIGURE 3:**
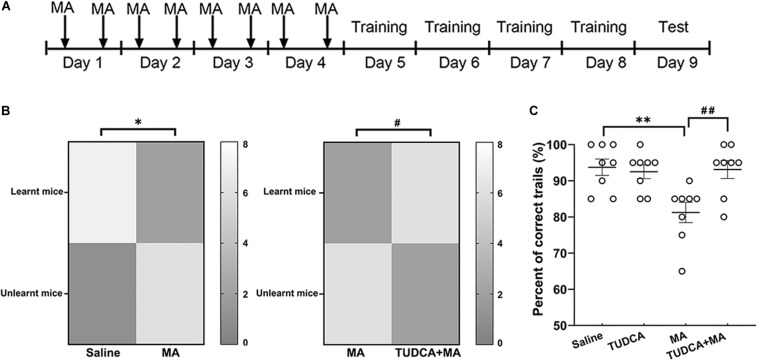
MA-induced impairment of recognition memory is protected by TUDCA in electro-stimulus Y-maze. **(A)** The experimental flow diagram of electro-stimulus Y-maze task. **(B)** The number of mice exhibiting learnt and unlearnt in different groups. The present results indicated that a lower percent of MA-treated mice exhibited learnt in comparison with the saline group, whereas the percentage of mice exhibiting learnt in the TUDCA + MA group was higher than that of the MA group. **(C)** The percentage of correct trials in MA group was lower than that of the Saline group, while mice of the TUDCA + MA group represented a higher percent of correct trials in comparison with the MA group. *p < 0.05, **p < 0.01 vs. the Saline group. ^#^*p* < 0.05, ^##^*p* < 0.01 vs. the MA group. Data was presented as the mean ± SEM, *n* = 8 per group.

Electro-stimulus Y-maze task was performed (*n* = 8 per group) to evaluate the effect of drug administration on the formation of recognition memory. The apparatus was manufactured by Zhangjiagang Biomedical Equipment Manufacturing Co., Ltd, and consisted of 3 arms with equal dimensions (65 × 15 × 15 cm, length × width × height), two of which were arranged at 120° angles. At the bottom of each arm, there were conductive gratings that were 0.5 cm in diameter and 0.5 cm in space. Each arm contained a light source. Three seconds after a light was turned on in one arm, an electrical stimulation lasting 10 s was given in the other two arms as well as the junctional zone. The task was performed as previously reported (Yu et al., 2003) with the procedure modified according to our experimental design. Mice were initially administered i.p. injections of drugs for 4 days, followed by 4 days training with 20 trials each day (Figure 3A). When performing the trials, electrical stimulation was given at random in the three arms and the junctional zone to help mice learn and remember that the arm in which the light was on was a safe area. If a mouse escaped into the safe arm within 10 s following electrical stimulation, the response would be recognized as correct. The testing, which consisted of 20 trials of electrical stimulation, was performed 24 h after the 4 days of training. For each mouse, 18 or more correct responses within 20 trials indicated learnt. Indices to evaluate the recognition memory included the percentages of correct trials and mice exhibiting learnt.

### Novel Object Recognition

**FIGURE 4:**
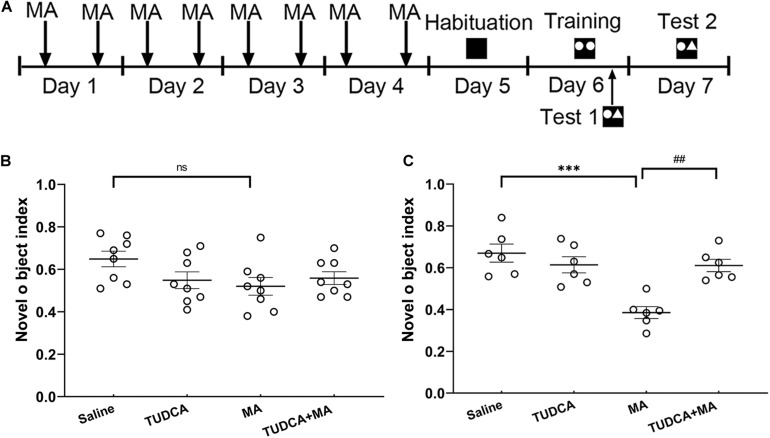
MA-induced long-term recognition memory loss in novel object recognition task is avoided by TUDCA. **(A)** The experimental flow diagram of NOR task. **(B)** In Test 1, there was no difference in the novel object index (NOI) between the four groups. **(C)** In Test 2, MA-treated mice exhibited a lower NOI, compared with that of the Saline group, TUDCA + MA-treated mice exhibited a higher NOI in comparison with that of the MA group. ***p < 0.001 vs. the Saline group. ^##^*p* < 0.01 vs. the MA group, ns indicated *p* > 0.05. Data was expressed as the mean ± SEM, *n* = 8 per group.

Novel object recognition (NOR) task was performed using a Plexiglas box (40 × 40 × 40 cm, length × width × height), manufactured by Shanghai Jiliang Software Technology Co., Ltd. The task consisted of three processes: habituation, training and testing. During the habituation (day 5), there was no object in the arena and mice were allowed to move freely for 20 min to become familiar with the environment. During the training (day 6), two identical objects were placed in the arena and mice were allowed to explore the objects for 10 min. During the testing, one of the objects was replaced with a novel object and mice were placed in the arena for 7 min to explore the two objects. In the present study, Test 1 and Test 2 were performed 30 min and 24 h post the training, respectively (Figure 4A). Novel object index (NOI), which is the time spent exploring the novel object divided by the time spent exploring the two objects, was used to evaluate the formation of recognition memory.

### Western Blotting

Mice were killed by an overdose of isoflurane 24 h after the last ingestion of MA and the hippocampal tissue was dissected on ice to extract the whole protein fraction. Western blotting was performed as previously reported (Cai et al., 2016) to examine the expression of ER stress marker proteins, including binding immunoglobulin protein (Bip) (Abcam, ab21685), cyclic AMP–dependent transcription factor (ATF)-4 (Abclonal, A8687), phosphorylated eukaryotic translation initiation factor 2α (p-eIF2α) (CST, 3398s), ATF-6 (Abclonal, A0202) and CCAAT-enhancer binding protein homologous protein (Chop) (Abclonal, A0221). Values of these proteins were normalized to that of actin (Applygen, C1313). Bands were semi-quantified using Image J software (NIH).

### Electrophysiology

The effect of MA on the induction of LTP *in vivo* of the perforant path (PP)–dentate gyrus (DG) pathway in the hippocampus was investigated as previously described (Gureviciene et al., 2004). Mice (*n* = 5 per group) were anesthetized with urethane (1.5 g/kg, i.p.) and then a pair of stimulating electrodes were implanted into the perforant path of the left hemisphere at A/P: −3.8 mm, M/L: −3.0 mm, D/V: −1.5 mm (from the dura). Meanwhile, a pair of recording electrodes were implanted into the dentate gyrus of the left hemisphere at A/P: −2.0 mm, M/L: −1.4 mm, D/V: −1.5 mm (from the dura, Figure 6A). The population spike (PS) was induced using monopolar pulses (duration, 400 μs; frequency, 1/30 Hz) using an Isolated Pulse Stimulator (A-M SYSTEMS Co., Ltd.) The PS was reported using a Differential AC Amplifier (A-M SYSTEMS Co., Ltd.) and Axon Digidata 1550A Data Acquisition System (Molecular Devices Co., Ltd.). When the stabilized PS (Figure 6B) lasted at least 30 min, we regulated the stimulating current to yield a PS that was 30–50% of the maximum amplitude. The PS was recorded for 30 min and the amplitude of PS was homogenized as the baseline. Then the HFS, consisting of three trains of 10 bursts (duration, 400 μs; frequency, 300 Hz) with an interval of 10 s between each train, was used to induce LTP *in vivo*. Next, the PS was yielded using formerly single monopolar pulses and recorded for 60 min. The amplitude of PS was calculated using pClamp10.0 software (Molecular Devices Co., Ltd.; Figure 6C).

### Statistical Analysis

All experiments were randomized and performed in a blinded manner. Data was presented as the mean ± SEM. Statistical analysis was performed using GraphPad Prism 8.0 (GraphPad Software, Inc.). For EPM testing, a one-way ANOVA was used to analyze the difference in the percent time spent in the open arms. For MWM testing, a two-way repeated-measures ANOVA followed by Bonferroni’s multiple comparison test was used to analyze the difference in escaped latency and a one-way ANOVA followed by Bonferroni’s multiple comparison test was used to analyze the difference in crossing latency and platform site crossings. For electro-stimulus Y-maze testing, a chi-square test was used to analyze the difference in the percentage of mice exhibiting learnt; a one-way ANOVA followed by Bonferroni’s multiple comparison test was used to analyze the percentage of correct trials. For NOR testing, a one-way ANOVA followed by Bonferroni’s multiple comparison test was used to analyze the difference in NOI. For electrophysiological experiments, a paired *t*-test was used to determine the difference in PS amplitude before and post HFS between different groups. The level of statistical significance was set at *p* < 0.05.

## Results

### MA Administration Has No Effect on the Anxiety-Like Behavior of Mice

The present results showed that there was no significant difference (*p* > 0.05) in the percent time spent in the open arms between the four groups (Figure 1B). Therefore, 4 days i.p. injections of MA had no effect on the tension and anxiety of mice.

### TUDCA Pre-treatment Ameliorates the Impairment of Spatial Memory Caused by MA

In the MWM testing, a two-way repeated-measures ANOVA of escaped latency revealed significant effects of time [*F*_(__3,36__)_ = 74.38, *p* < 0.001] and groups [*F*_(__3,36__)_ = 7.848, *p* < 0.001], but not their interaction [*F*_(__9,108__)_ = 0.684, *p* > 0.05]. MA-treated mice exhibited increased escaped latencies on day 1 (*p* < 0.05), day 2 (*p* < 0.01), and day 4 (*p* < 0.05) of the training session, compared with that of saline-treated mice; however, there was no difference in the escaped latency between the TUDCA + MA and Saline groups. Moreover, the escaped latency of TUDCA + MA-treated mice was less than that of MA group on the fourth day of training (*p* < 0.05; Figure 2B). In the probe test, MA-treated mice exhibited an increased crossing latency (*p* < 0.01) and decreased platform site crossings (*p* < 0.001) in comparison with saline-treated mice, whereas mice of the TUDCA + MA group exhibited a decreased crossing latency (*p* < 0.05) and increased platform site crossings (*p* < 0.05), compared with MA-treated mice (Figures 2D,E). Meanwhile, no difference in the swimming speed was found between the four groups (*p* > 0.05) in the probe test (Figure 2C).

### MA-Induced Recognition Memory Defects Are Rescued by TUDCA Pre-treatment

In the electro-stimulus Y-maze testing, it was found that the percentage of mice exhibiting learnt in the MA group was lower than that of the Saline group (*p* < 0.05), meanwhile, the percentage of correct trials in the MA group was lower than that of the Saline group (*p* < 0.01). An increased percentage of TUDCA + MA-treated mice exhibited learnt (*p* < 0.05), compared with the MA group. The percentage of correct trials in the TUDCA + MA group was also higher than that of the MA group (*p* < 0.01; Figures 3B,C). In the NOR testing, no significant difference in NOI was found between the four groups in Test 1 (*p* > 0.05; Figure 4B). However, in Test 2, the NOI of the MA group was lower than that of the Saline group (*p* < 0.001). Mice of the TUDCA + MA group exhibited an increased NOI, compared with the MA group (*p* < 0.01; Figure 4C).

### MA-Induced ER Stress Is Inhibited by TUDCA Pre-treatment

**FIGURE 5:**
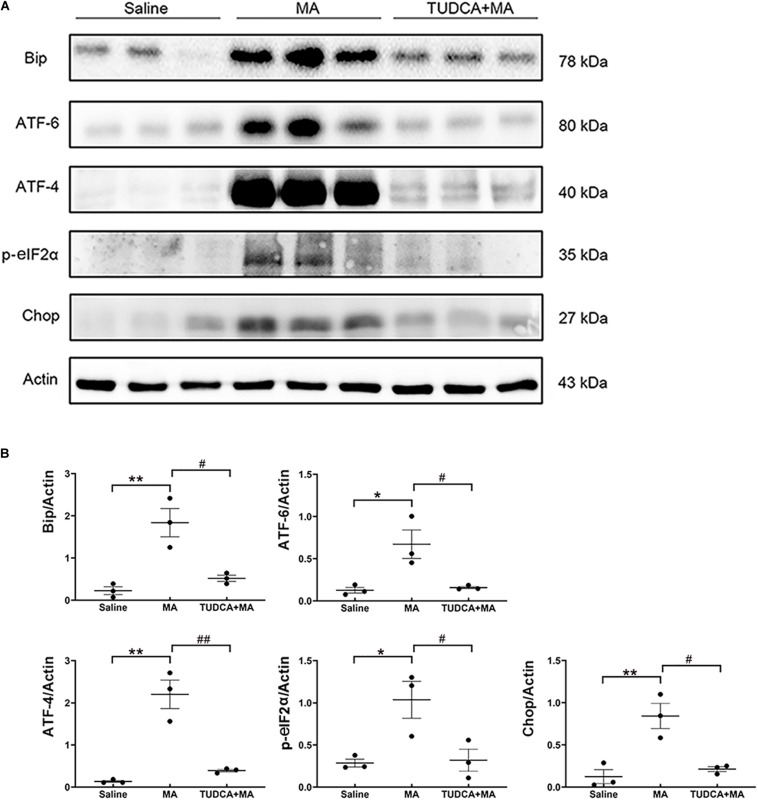
MA-induced ER stress is reversed by TUDCA pre-treatment. **(A,B)** Four-day injections of MA increased the expression levels of Bip, ATF-4, ATF-6, p-eIF2α, and Chop, all of which were reversed by TUDCA pre-treatment. *p < 0.05, **p < 0.01 vs. the Saline group. ^#^*p* < 0.05, ^##^*p* < 0.01 vs. the MA group. Data was presented as the mean ± SEM, *n* = 3 per group.

The results of the Western blotting suggested that the administration of MA in the present study increased the expression levels of ER stress marker proteins, including Bip (*p* < 0.01), ATF-6 (*p* < 0.05), ATF-4 (*p* < 0.01), *p*-eIF2α (*p* < 0.05), and Chop (*p* < 0.01), all of which were reversed by TUDCA (Figures 5A,B).

### MA-Induced Inhibition of LTP *in vivo* Is Reversed by TUDCA Pre-treatment

**FIGURE 6:**
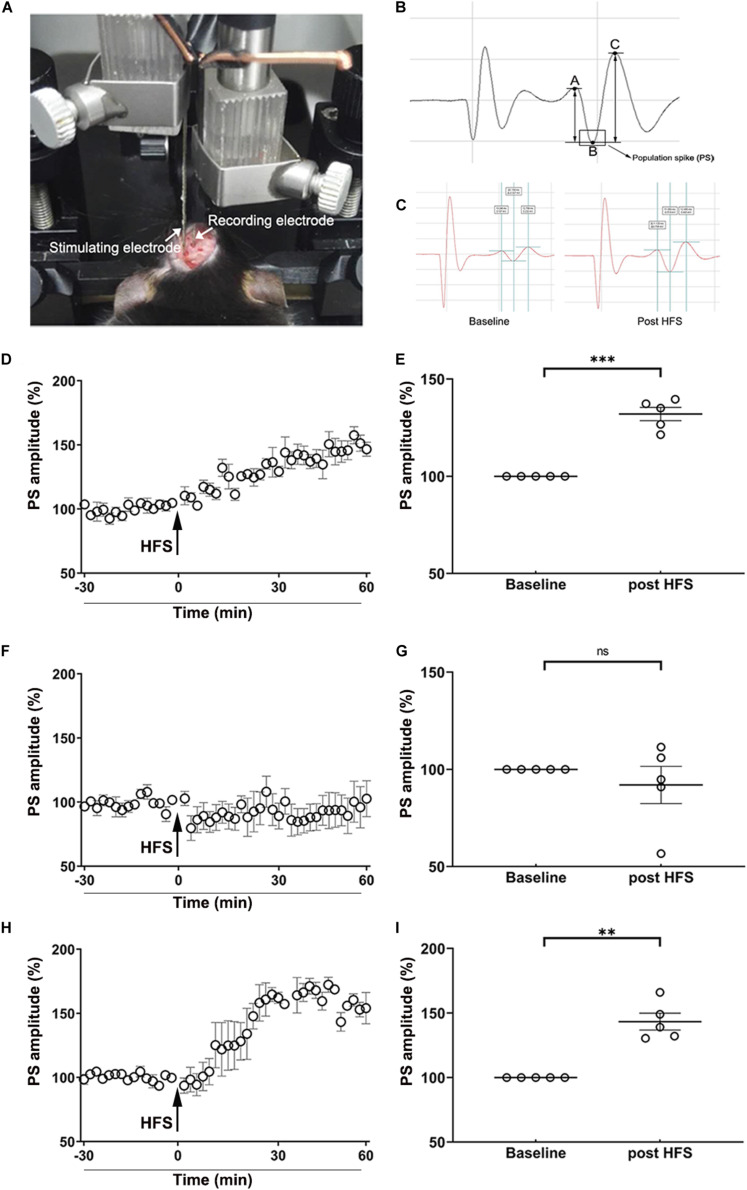
MA-induced inhibition of LTP *in vivo* is reversed by TUDCA pre-treatment. **(A)** The position of stimulating electrodes and recording electrodes in the electrophysiological experiments. **(B)** A typical form of the population spike (PS) was consisted of a descending branch (AB) and an ascending branch (BC). The amplitude of PS was calculated by the average of the potential difference of AB and BC. **(C)** A typical enhancement of PS amplitude post-HFS, compared with the baseline. **(D,E)** The HFS induced a significant increase of the PS amplitude in the Saline group. **(F,G)** When mice were treated with MA, there was no difference in the PS amplitude post-HFS in comparison with the baseline. **(H,I)** When mice were pretreated with TUDCA, the HFS induced a prominent enhancement of the PS amplitude. **p < 0.01, ***p < 0.001 vs. the baseline, ns indicated *p* > 0.05. Data was presented as the mean ± SEM, *n* = 5 per group.

The obtained data indicated that the HFS used in the present study induced LTP *in vivo* in the hippocampus, evidenced by the PS amplitude of saline-treated mice was enhanced to 132.00 ± 3.43% of the baseline (*p* < 0.001) post-HFS (Figures 6D,E). After the mice were administered i.p. injections of MA, however, the amplitude of PS could not be enhanced by the HFS (*p* > 0.05; Figures 6F,G). When mice were pre-treated with TUDCA, the PS amplitude was increased to 143.30 ± 6.56% of the baseline (*p* < 0.01) (Figures 6H,I).

## Discussion

MA is a worldwide abused illicit drug (Centazzo et al., 2019; Xu and Liu, 2019) and MA-induced cognitive impairment has been of increasing concern to the public (Morgan et al., 2012). Recently, several potential mechanisms underlying MA-induced neural damage have been proposed, including hyperthermia, excitotoxicity, inflammation, mitochondrial dysfunction, and oxidative stress (Yamamoto et al., 2010; Moszczynska and Callan, 2017). ER stress, which is involved in a variety of diseases, has been given much attention and considered as one mechanism mediating MA-induced neurotoxicity. Nevertheless, studies regarding the association between ER stress and MA-induced neural damage have largely focused on molecular mechanisms rather than behavioral manifestation. According to the molecular toxicology studies in our laboratory and other laboratories, it is postulated that ER stress may be one mechanism underlying the cognitive dysfunction caused by MA. To verify this hypothesis, the influence of MA administration on the memory formation was studied using different behavioral tests, including the MWM, electro-stimulus Y-maze, and NOR tasks. EPM testing was first performed to evaluate the effect of MA on anxiety-like behavior in mice. Compared with saline-treated mice, mice administered i.p. injections of MA exhibited no difference in the percentage of time spent in open arms, indicating that MA ingestion did not affect anxiety-like behavior of mice. MWM task was performed to investigate whether ER stress was involved in MA-induced spatial memory impairment. Consistent with the findings of previous reports (Vorhees and Williams, 2006; Heysieattalab et al., 2016), MA induced spatial memory impairment, as indicated by the increased escaped latencies during the training stage, crossing latency as well as fewer platform site crossings in the probe test. Compared with MA-treated mice, TUDCA + MA-treated mice exhibited decreased escaped latency, crossing latency as well as more platform site crossings, demonstrating that inhibiting ER stress rescued MA-induced impairment of spatial memory. Besides spatial memory, we also evaluated the effect of MA exposure on recognition memory by conducting electro-stimulus Y-maze and NOR tasks. The present results of these two experiments suggested that MA-induced inhibition of recognition memory could also be protected by TUDCA pre-treatment. Meanwhile, we evaluated the effect of MA administration on the expression levels of ER stress marker proteins in the hippocampus, such as Bip, ATF-6, ATF-4, p-eIF2α, and Chop. The results showed that MA increased the expression levels of these proteins, while TUDCA pre-treatment reversed the effect of MA, confirming that the subacute administration of MA induced cognitive impairment via ER stress in the present study.

In the NOR tasks, two different tests were conducted to distinguish the effect of MA ingestion on working memory and long-term memory. In Test 1, MA-treated mice did not show a decreased NOI, while MA-treated mice had a lower NOI in Test 2, indicating 4 days injections of MA impaired long-term recognition memory, but had no effect on short-term recognition memory. It has been generally accepted that memory can be divided into two different forms, short-term memory (STM) and long-term memory (LTM) (Scoville and Milner, 1957), which have different duration as well as molecular mechanisms. Previous studies have suggested that newly synthesized proteins are essential for the formation of LTM rather than STM (Davis and Squire, 1984). Recently, researchers have further found that increased expression level of p-eIF2α impairs the induction of long-lasting LTP (L-LTP) and consolidation of spatial memory, indicating that translational control of gene expression by eIF2α signaling pathway may be a molecular switch for LTM formation (Costa-Mattioli and Sonenberg, 2008). Enhanced phosphorylation of eIF2α serves a negative control of the gene expression and protein synthesis (Dever, 2002) and p-eIF2α is also involved in the ER stress. In the present study, MA administration increased the phosphorylation of eIF2α and the expression level of ATF-4, which is a downstream of p-eIF2α. It was speculated that the inhibition of gene translation caused by p-eIF2α may be the reason why MA ingestion disturbed the formation of LTM, but not STM.

At last, the effect of MA poisoning on synaptic plasticity in the PP-DG pathway of the hippocampus was investigated to study the underlying mechanisms for MA-induced memory loss. We selected LTP *in vivo*, an intensively studied cellular model of the memory, to further study the role of MA-induced ER stress in the disruption of memory acquisition. LTP, referring to a sustained increase in efficiency of synaptic transmission caused by trains of high-frequency stimulation, was first fully described by Bliss and Lomo (1973). A great deal of research has revealed that LTP may be a biological substrate for at least some forms of memory (Lynch, 2004). In the present study, we found that MA-induced inhibitory of LTP in the hippocampus could also be reversed by ER stress inhibitor, TUDCA, indicating that ER stress may be a reason why LTP induction was inhibited by MA ingestion. However, the detailed mechanism for MA restraining LTP *in vivo* through ER stress needs further investigation in the future studies. In conclusion, MA inhibited long-term memory acquisition and synaptic plasticity by evoking ER stress.

## Data Availability Statement

The raw data supporting the conclusions of this article will be made available by the authors, without undue reservation, to any qualified researcher.

## Ethics Statement

The animal study was reviewed and approved by the Animal Care and Use Committee of the Beijing Institute of Pharmacology and Toxicology.

## Author Contributions

GC conducted all of the experiments with the help of GY and ZY and wrote the manuscript with the help of XW. LT and RS designed the research. XX analyzed the results. All authors contributed to the article and approved the submitted version.

## Conflict of Interest

XW was employed by Becton, Dickinson and Company, Guangzhou, China. The remaining authors declare that the research was conducted in the absence of any commercial or financial relationships that could be construed as a potential conflict of interest.
